# A blood-based miRNA signature for early non-invasive diagnosis of preeclampsia

**DOI:** 10.1186/s12916-022-02495-x

**Published:** 2022-09-13

**Authors:** Siqi Bao, Tong Zhou, Congcong Yan, Jiale Bao, Fan Yang, Shan Chao, Meng Zhou, Zhangye Xu

**Affiliations:** 1 Department of Gynecology and Obstetrics, The Second Affiliated Hospital and Yuying Children’s Hospital of Wenzhou Medical University, Wenzhou, 325027 People’s Republic of China; 2 School of Biomedical Engineering, Wenzhou Medical University, Wenzhou, 325027 People’s Republic of China; 3 Institutes for Shanghai Pudong Decoding Life, Shanghai, People’s Republic of China

**Keywords:** Biomarker, Early diagnosis, Liquid biopsy, MicroRNA, Non-invasive, Preeclampsia

## Abstract

**Background:**

Preeclampsia (PE) is a multisystemic maternal syndrome with substantial maternal and fetal morbidity and mortality. Currently, there is no clinically viable non-invasive biomarker assay for early detection, thus limiting the effective prevention and therapeutic strategies for PE.

**Methods:**

We conducted a discovery–training–validation three-phase retrospective and prospective study with cross-platform and multicenter cohorts. The initial biomarkers were discovered and verified in tissue specimens by small RNA sequencing and qRT-PCR. A miRNA signature (miR2PE-score) was developed using Firth’s bias-reduced logistic regression analysis and subsequently validated in two independent multinational retrospective cohorts and two prospective plasma cohorts.

**Results:**

We initially identified five PE-associated differentially expressed miRNAs from miRNA sequencing data and subsequently validated two miRNAs (*miR-196b-5p* and *miR-584-5p*) as robust biomarkers by association analysis with clinical characteristics and qRT-PCR in tissue specimens in the discovery phase. Using Firth’s bias-reduced logistic regression analysis, we developed the miR2PE-score for the early detection of PE. The miR2PE-score showed a high diagnostic performance with an area under the receiver operating characteristic curve (AUROC) of 0.920, 0.848, 0.864, and 0.812 in training, internal, and two external validation cross-platform and multicenter cohorts, respectively. Finally, we demonstrated the non-invasive diagnostic performance of the miR2PE-score in two prospective plasma cohorts with AUROC of 0.933 and 0.787. Furthermore, the miR2PE-score revealed superior performance in non-invasive diagnosis compared with previously published miRNA biomarkers.

**Conclusions:**

We developed and validated a novel and robust blood-based miRNA signature, which may serve as a promising clinically applicable non-invasive tool for the early detection of PE.

**Graphical Abstract:**

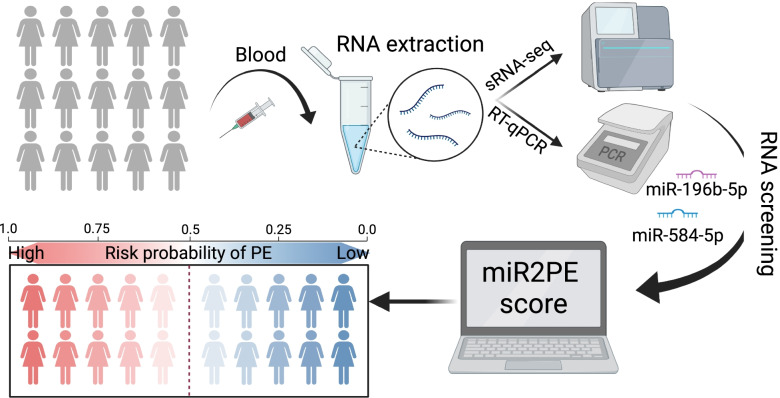

**Supplementary Information:**

The online version contains supplementary material available at 10.1186/s12916-022-02495-x.

## Background

Preeclampsia (PE) is a multisystemic disease characterized by hypertension [1], endothelial cell dysfunction [2], poor extravillous cytotrophoblast invasion [3], and proteinuria and fetal growth retardation [4] after 20 weeks of gestation. This multisystem disorder affects approximately 5–8% of pregnancies [5, 6] and is one of the most severe pregnancy complications that pose a risk to both mother and baby. Each year, 70,000 women and 50,000 babies approximately die from this disorder worldwide [7].

Once PE occurs, the treatment available is limited to symptomatic treatment and the PE progression will not be effectively prevented, leading to severe maternal and fetal complications. Therefore, predicting PE in the early stage of disease, before symptoms present, is warranted to prevent or reduce the frequency and severity of PE. However, early diagnosis and effective treatment of PE remain a challenge as diagnostic criteria for PE are non-specific signs and symptoms, and its severity criteria are poorly correlated with adverse maternal and fetal outcomes. Over the past few years, there have been increasing efforts to improve the early diagnosis and treatment of PE. Angiogenesis-related factors, such as soluble fms-like tyrosine kinase-1 (sFlt-1), placental growth factor (PlGF), and soluble endoglin (sEng), could be used to predict PE [8–10]. However, diabetes mellitus, parity, twins, and advanced maternal age influenced the definition of normal parameters for these markers leading to higher variation in cutoff values and predictive efficacy across different ethnic and geographical populations [11–13]. However, despite these efforts, none of these biomarkers is adequate and satisfactory in managing PE. Therefore, there is an urgent need to identify clinically viable biomarkers and tools for the early diagnosis and personalized intervention of PE.

MiRNAs are single-stranded (~ 22 nucleotides), non-coding RNAs responsible for the mechanism of posttranscriptional gene expression regulation [14–17]. MiRNAs are characterized by a long half-life and high stability, and their stability is 10 times higher than that of mRNAs [18]. During pregnancy, placental trophoblast cells at the maternal–fetal interface produce a large number of miRNAs, and its expression level changes with the increase of gestational age and placental development, which highlights its importance in placental regulation. MiRNAs are highly stable in serum, plasma, and urine, making miRNA possible as non-invasive biomarkers for detecting and diagnosing PE [19]. Increasing evidence indicated the potential roles of miRNAs in the pathogenesis and treatment of PE [20, 21]. For example, a recent study has shown that *miR-27a* is overexpressed in PE placenta, and inhibition of *miR-27a* may be a probable treatment for PE [22]. Several miRNAs, such as *Let-7a*, *miR-125b*, *miR-133b*, *miR-384*, *miR-101*, *miR-206*, and *miR-203a-3p*, have been recently identified as candidates that may be involved in the progression or suppression of PE [22–26]. However, the predictive value and clinical translation potential of miRNA for early diagnosis of PE remain uncertain and need further investigation.

In this study, we conducted a discovery–training–validation three-phase multicenter study with cross-platform retrospective and prospective cohorts to identify and validate a blood-based miRNA signature for early non-invasive diagnosis of PE.

## Methods

### Ethics statement

This study was approved by the human ethics committee at the Second Affiliated Hospital and Yuying Children’s Hospital of Wenzhou Medical University (approval number: LCKY2020-242), and informed consent was obtained from all patients and their families. And the study was performed in accordance with the regulations and guidelines established by this committee.

### Study design and patient cohorts

**Fig. 1:**
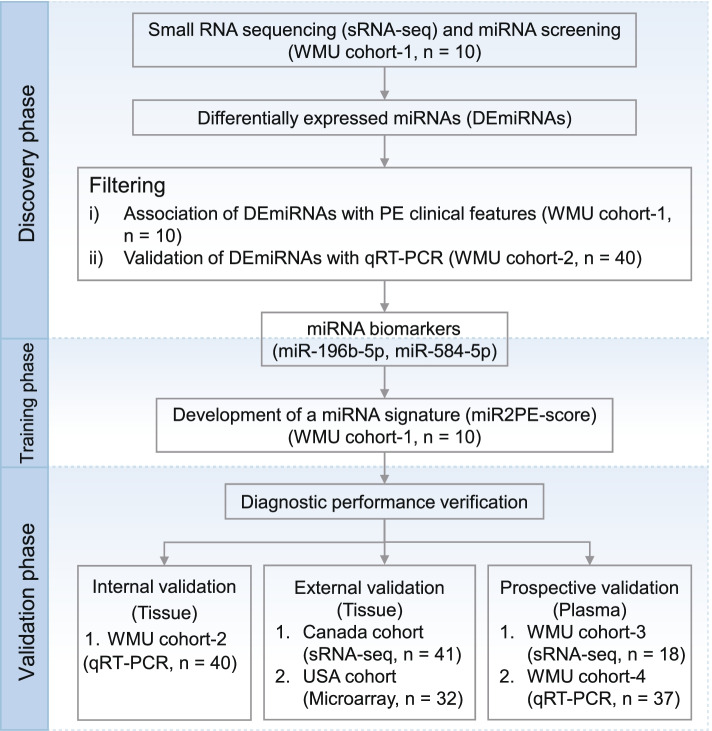
Study flowchart. A discovery–training–validation three-phase study was conducted with multicenter and cross-platform retrospective and prospective cohorts including four in-house cohorts (WMU cohorts 1–4), the Canada cohort, and the USA cohort

This study was carried out in three phases: discovery, training, and validation. The details of the overall study are shown in Fig. 1. In the discovery phase, we conducted miRNA sequencing in a retrospective cohort (WMU cohort 1) for genome-wide screening of candidate miRNA biomarkers and then verified the expression levels of the candidate miRNA biomarkers in 20 PE patients and 20 healthy controls from WMU cohort 2 using qRT-PCR for identifying robust miRNA biomarkers. In the training phase, a miRNA signature for early diagnosis of PE (miR2PE-score) was developed in WMU cohort 1. In the validation phase, the performance of miR2PE-score was firstly evaluated in two independent multinational cohorts (Canada cohort and USA cohort) from the Gene Expression Omnibus (GEO) database (https://www.ncbi.nlm.nih.gov/geo/). The Canada cohort (https://www.ncbi.nlm.nih.gov/geo/query/acc.cgi?acc=GSE114349) contained 21 normotensive women and 20 preeclamptic women [27], and the USA cohort (https://www.ncbi.nlm.nih.gov/geo/query/acc.cgi?acc=GSE84260) [28] included 16 normotensive women and 16 preeclamptic women, respectively. To further validate the non-invasive diagnostic performance, the miR2PE-score was further tested in two prospective plasma cohorts, including WMU cohort 3 with 10 PE patients and 8 healthy controls and WMU cohort 4 with 19 PE patients and 18 healthy controls, respectively.

### Sample collection and preparation

We collected the samples at the Department of Obstetrics, the Second Affiliated Hospital and Yuying Children’s Hospital of Wenzhou Medical University (Zhejiang, China) between June 2020 and December 2021. Blood samples were obtained from pre-cesarean women with overnight (≈10 h) fasting in 10-ml EDTA-coated Vacutainer tubes before any medical interventions. Five milliliters of blood for obtaining plasma was separated by centrifugation at 3000 rpm for 15 min at 20℃ within 30 min after each collection, and then the supernatants were extracted to a new tube.

Leukocytes were purified from another 5 ml blood using red blood cell lysis solution (Solarbio, China) following the manufacturer’s protocol. In brief, 5 ml blood was incubated with a threefold volume of red cell lysate on ice for 15 min and mixed evenly. The mixture was centrifuged at 450xg for 10 min at 4℃, the supernatant was discarded, and the leukocyte precipitate was obtained. The experiment described above was repeated three times. Finally, red cells were dissolved and leukocytes were washed with PBS.

Placental samples were rapidly processed as previously described [29]. In brief, the tissue was sectioned transversally (~ 5 cm) near the cord insertion site. Both the decidual layer along with the basal plate and the chorionic surface and membranes were removed by dissection. Each sample was rinsed thoroughly in cold physiological saline solution and frozen in liquid nitrogen.

All samples were stored at − 80 °C freezer, where aliquots were stored until assay.

### MiRNA discovery and quantification by next-generation sequencing (NGS)

A total amount of 1 μg total RNA per sample was used as input material for the small RNA library. Sequencing libraries were generated using TruSeq® miRNA Sample Prep Kit for Illumina®. Manufacturers’ recommendations and index codes were added to attribute sequences to each sample. The first-strand cDNA was synthesized using SuperScript II (ThermoFisher), and library quality was assessed on the Qubit system (Invitrogen). Raw data preparations were sequenced on a Nova6000 platform (Illumina). Fastp (0.20.1) was applied to remove low-quality reads and adapters of the raw sequencing reads. Using the miRdeep2 mapper.pl command, the filtered clean sequencing reads were mapped to human genome GRCh38 obtained from ENSEMBL (release 95). Then, the miRDeep2.pl command was executed to quantify known miRNAs, with miRNA reference files containing mature miRNA sequences and their corresponding precursor sequences downloaded from the miRBase database (https://www.mirbase.org/).

### Quantitative reverse transcription polymerase chain reaction (qRT-PCR)

qRT-PCR analysis was used to verify miRNA expression. RNA was isolated from tissues and plasma using the miRcute miRNA Isolation Kit (Tian Biotech, China) and reverse transcribed using PrimeScript RT Master Mix for RT-PCR (Roche Diagnostics, USA) according to the manufacturer’s protocol. For miRNA expression analysis, miRNA quantification was determined by using Bulge-Loop™ miRNA qRT-PCR Primer Sets (one RT primer and a pair of qRT-PCR primers for each set) specific for *miR-196b-5p* and *miR-584b-5p*, designed by RiboBio (RiboBio, China). All reagents for stem-loop RT mature miRNA assays were obtained from RiboBio (RiboBio, China). Real-time PCR was performed using SYBR Select Rremix Ex Taq II (TaKaRa Bio, Japan) on the Bio-Rad CFX96 Real-Time Detection System (Bio-Rad, USA). Data were normalized to levels of U6 (tissue) or cel-miR-39-3p (plasma) as appropriate, respectively. All analysis was carried out using the 2^−ΔCt^ method.

### Development of the miRNA signature for early diagnosis of PE

Differential expression analysis of miRNAs was conducted between PE placental samples and normal placental samples using R package DESeq2 (V1.34.0) with the Wald test function. Those miRNAs with a fold change value greater than 2.0 or lower than 0.5 and a false discovery rate (FDR)-adjusted *p*-value lower than 0.05 were considered significantly differentially expressed miRNAs (DEmiRNAs). A Pearson correlation analysis was performed to filter DEmiRNAs related to clinical characteristics. To validate the sRNA-seq data, DEmiRNAs between the PE and normal groups were investigated by qRT-PCR in WMU cohort 2. Finally, the filtered DEmiRNAs were used to establish a miRNA signature (miR2PE-score) for PE diagnosis using Firth’s bias-reduced logistic regression model. The miR2PE-score limits the risk score to 0–1, and the meaning of that is the perceived probability of having PE according to our model. Using 0.5 as the threshold, samples with miR2PE-score > 0.5 are considered to have a high risk of PE, and vice versa.

### Functional enrichment analysis

The target genes of miRNAs were extracted from miRTarBase (https://mirtarbase.cuhk.edu.cn) [30], with at least one experimental verification as the standard. Gene Ontology (GO) term and Kyoto Encyclopedia of Genes and Genomes (KEGG) pathway enrichment analysis was performed using Metascape (https://metascape.org/) [31]. All genes of the human genome were used as the enrichment background. Terms and pathways with *p*-value < 0.05, the number of genes enriched greater than 2, and enrichment factor > 1.5 were considered enriched terms and pathways. According to semantic similarity, similar enriched GO terms or KEGG pathways with kappa score > 0.3 were clustered to form a functional network. Each node in the functional network represents a GO term or KEGG pathway. The node size is proportional to the number of overlapping genes between the genes of interest and the genes belonging to the term or pathway, and the node color represents a cluster annotation. Similar term or pathway nodes are marked with the same color and entries of the representing nodes are used as cluster annotations.

### Statistical analysis

All statistical analysis and generation of figures were performed with the statistical software R, version 4.0.5 (https://www.r-project.org), together with R packages “ggplot2,” “ggrepel,” “ggpubr,” “pheatmap,” “corrplot,” “pROC,” “logistf,” “caret,” and “PRROC.” The Mann–Whitney *U* test was applied to compare outcomes between two independent groups. The area under the receiver operating characteristic curve (AUROC), precision–recall (PR) curve, sensitivity (TPR), specificity (TNR), positive predictive value (PPV), negative predictive value (NPV), and overall accuracy (ACC) were used to evaluate the diagnostic performance.

## Results

### Characteristics of study populations

The clinical and demographic data were obtained during routine visits and were recorded using standard datasheets. Gestational time was calculated with an algorithm based on a participant’s last normal menstrual period confirmed by early ultrasound. Patients with PE were diagnosed according to the technical bulletin of the American College of Obstetricians and Gynecologists [32]. All PE patients were nulliparous or multigravida (range 1–2). Subjects were excluded if they had abnormal 1-h glucose tolerance tests or pre-existing medical conditions such as hypertension, cardiovascular disease, diabetes mellitus, renal disease, or other chronic systemic diseases.

Maternal plasma samples and placental tissues were collected as part of four cohorts. The clinical characteristics of each cohort according to women with and without PE are presented in Table 1. Maternal age and BMI were statistically similar between healthy controls and PE patients (all *p* > 0.05). As expected, in all cohorts, preoperative systolic blood pressure (PreSBP), preoperative diastolic blood pressure (PreDBP), and urine protein/urine CERA (mg/mmol) in patients with PE were significantly higher than those in healthy controls (all *p* < 0.05). There was an earlier median gestational age of delivery and lower mean birthweight in PE patients compared to healthy controls. Except for cohort 1, despite large variations among samples, albumin and blood urea nitrogen were statistically significant in the analysis with the other three cohorts included, respectively.

**Table 1:** Clinical characteristics of patients in four in-house cohorts

Characteristics	WMU cohort 1			WMU cohort 2			WMU cohort 3			WMU cohort 4		
	**Healthy controls (*n* = 5)**	**PE patients (*n* = 5)**	***p-value***	**Healthy controls (*n* = 20)**	**PE patients (*n* = 20)**	***p-value***	**Healthy controls (*n* = 8)**	**PE patients (*n* = 10)**	***p-value***	**Healthy controls (*n* = 18)**	**PE patients (*n* = 19)**	*p-value*
Mean maternal age (years)	30.2 ± 5.7	31.0 ± 6.1	0.84	29.8 ± 5.1	30.4 ± 4.5	0.69	34.8 ± 4.2	31.1 ± 5.1	0.12	30.9 ± 4.8	31.3 ± 4.5	0.78
Mean BMI (kg/m^2^)	28.8 ± 2.9	30.6 ± 5.5	0.53	27.8 ± 1.9	28.0 ± 3.2	0.85	25.2 ± 2.5	27.6 ± 4.2	0.18	27.0 ± 2.7	27.8 ± 3.7	0.50
PreSBP (mmHg)	115.0 ± 7.0	164.6 ± 21.5	< 0.01	117.6 ± 10.8	146.4 ± 17.2	< 0.001	117.1 ± 10.1	152.9 ± 20.8	< 0.001	117.4 ± 9.8	150.8 ± 17.7	< 0.001
PreDBP (mmHg)	70.0 ± 6.3	107.2 ± 10.0	< 0.001	72.0 ± 8.7	96.0 ± 13.1	< 0.001	69.8 ± 5.3	100.6 ± 12.3	< 0.001	72.2 ± 6.5	100.4 ± 11.0	< 0.001
Mean ALB (g/L)	33.3 ± 3.2	29.8 ± 4.3	0.18	37.0 ± 3.4	30.4 ± 6.6	< 0.001	35.6 ± 3.1	31.3 ± 4.9	< 0.05	36.4 ± 2.8	32.5 ± 4.7	< 0.01
Mean ALT (IU/L)	10.2 ± 3.9	20.6 ± 11.1	0.08	10.5 ± 5.0	14.5 ± 7.3	0.05	9.6 ± 3.6	16.8 ± 12.7	0.14	10.2 ± 6.0	20.1 ± 11.6	< 0.001
Mean BUN (mmol/L)	2.9 ± 0.6	4.4 ± 1.9	0.15	3.0 ± 0.8	4.1 ± 1.5	< 0.01	3.1 ± 0.8	5.2 ± 2.9	0.07	3.0 ± 0.9	4.5 ± 2.2	< 0.05
BUN/CERA	0.07 ± 0.02	0.08 ± 0.03	0.40	0.07 ± 0.02	0.08 ± 0.02	0.12	0.08 ± 0.03	0.10 ± 0.04	0.39	0.07 ± 0.03	0.09 ± 0.03	0.14
Urine protein/urine CERA (mg/mmol)	12.3 ± 3.5	517.9 ± 203.1	< 0.001	11.9 ± 4.3	245.0 ± 307.6	< 0.001	9.7 ± 3.9	508.4 ± 405.9	< 0.001	11.2 ± 3.2	295.0 ± 327.0	< 0.001
Median GA delivery (week)	38.2 ± 1.1	35.6 ± 2.9	0.10	38.4 ± 0.9	35.7 ± 2.6	< 0.001	37.9 ± 0.6	32.9 ± 4.8	< 0.05	38.5 ± 0.9	34.0 ± 4.1	< 0.001
No. preterm deliveries (%)	0 (0.0%)	3 (60.0%)	0.17	0 (0.0%)	12 (60.0%)	< 0.001	0 (0.0%)	7 (70.0%)	< 0.01	0 (0.0%)	12 (63.2%)	< 0.001
Mean birthweight (g)	3376.0 ± 405.3	2244.0 ± 968.8	< 0.05	3412.0 ± 352.5	2461.0 ± 689.8	< 0.001	3358.0 ± 343.3	1795.0 ± 860.1	< 0.001	3443.0 ± 432.8	2138.0 ± 848.3	< 0.001

Values are given as mean ± sd. or *n*(%)

*BMI* Body mass index during pregnancy, *PreSBP* Preoperative systolic blood pressure, *PreDBP* Preoperative diastolic blood pressure, *ALB* Albumin, *ALT* Alanine transaminase, *BUN* Blood urea nitrogen, *CREA* Creatinine, *GA* Gestational age

*p* values for comparisons between healthy controls with PE patients

### Identification and verification of PE-associated miRNA biomarkers

**Fig. 2:**
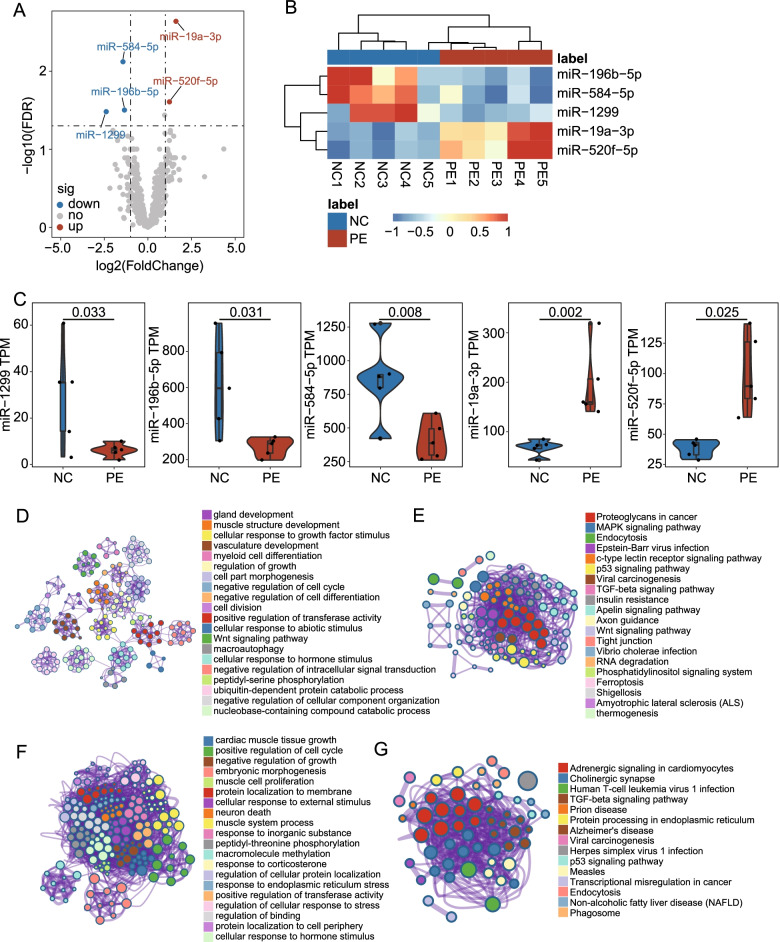
Identification of differentially expressed miRNAs. **A** Volcano plot of the differential expression analysis comparing PE placental samples and normal placental samples. The log2FC is shown on the *x*-axis and the − log10(FDR-adjusted *p*-value) is shown on the *y*-axis. Each dot is a miRNA in the analysis. The red dots are significantly up-regulated miRNAs. The blue dots are significantly down-regulated miRNAs. **B** Unsupervised clustering based on the expression pattern of 5 DEmiRNAs. The columns represent samples, and the rows represent miRNAs. **C** Boxplots of the expression value of 5 DE miRNAs of the PE and normal samples in WMU cohort 1. Horizontal lines: median values. Statistical analysis was performed using the Wald test. *p*-value was adjusted using the FDR method. Metascape enrichment network visualization of enriched GO BP terms (**D**) and KEGG pathways (**E**) of up-regulated miRNA target genes. Metascape enrichment network visualization of enriched GO BP terms (**F**) and KEGG pathways (**G**) of down-regulated miRNA target genes. DEmiRNA, differentially expressed miRNA; FC, fold change; FDR, false discovery rate; GO, Gene Ontology; BP, biological processes; KEGG, Kyoto Encyclopedia of Genes and Genomes

In the discovery stage, we first compared the expression profiles of 2888 miRNAs derived from miRNA sequencing between placental tissue samples of 5 PE patients and 5 healthy controls in WMU cohort 1 and identified five significantly differentially expressed miRNAs (DEmiRNAs) with the screening strategy of fold change > 2.0 or lower than 0.5 and FDR-adjusted *p* < 0.05 between the PE and the normal group (Fig. 2A). Unsupervised hierarchical clustering analysis showed that the expression patterns of five DEmiRNAs were able to discriminate PE samples from healthy controls (Fig. 2B). Among the five DEmiRNAs, two miRNAs (*miR-19a-3p* and *miR-520f-5p*) were found to be up-regulated and three miRNAs (*miR-584-5p*, *miR-196b-5p*, and *miR-1299*) were down-regulated in PE placental samples compared with normal placental samples (Fig. 2C). Functional enrichment analysis of GO and KEGG showed that the target genes of DEmiRNAs tended to be enriched in biological processes and pathways related to fetal growth and development and the pathogenesis of PE (Fig. 2D–G).

**Fig. 3:**
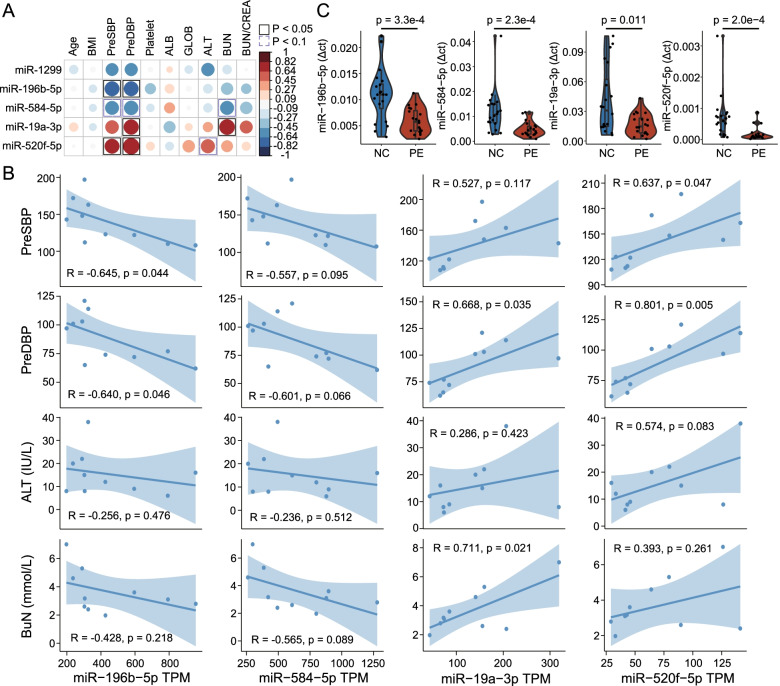
Identification of PE-associated miRNA biomarkers. **A** The visualization of the Pearson correlation analysis. The blue circle represents negative correlations. The red circle represents positive correlations. The black box represents Pearson correlation *p*-value < 0.05; the purple box represents Pearson correlation *p*-value < 0.1. **B** Scatter plots of the four DEmiRNA expression and clinical characteristics. **C** Boxplots showing the expression levels of four DEmiRNAs in the PE and normal samples in WMU cohort 2. Horizontal lines: median values. The statistical analysis was performed using the Mann–Whitney *U* test

To further examine the association between expression levels of DEmiRNAs and PE-associated clinical characteristics (including age, BMI during pregnancy, PreSBP, PreDBP, and the content of platelet, ALB, GLOB, ALT, BUN, and BUN/CREA), we calculated Pearson correlation coefficients and found that four of the five DEmiRNAs were associated with at least one PE-associated clinical characteristics (Fig. 3A, B). Thus, we further verified the expression of these four DEmiRNAs in placental tissue samples of 20 PE patients and 20 healthy controls from WMU cohort 2 using qRT-PCR and validated *miR-196b-5p* and *miR-584-5p* as reliable and robust biomarkers (Fig. 3C).

### Establishment and verification of a miRNA signature for PE diagnosis in two cross-platform retrospective cohorts

To establish a miRNA signature for the detection of PE, Firth’s bias-reduced logistic regression method was used to construct the predictive model based on the expression levels of *miR-196b-5p* and *miR-584-5p* in WMU cohort 1. Finally, a miR2PE-score was calculated for each sample using the observed weights from the regression model and the expression value of *miR-196b-5p* and *miR-584-5p* with the equation:

$$miR2PE-score=\frac{\ell^{-0.500\ast miR-196b-5p-1.227\ast miR-584-5p-0.055}}{1+\ell^{-0.500\ast miR-196b-5p-1.227\ast miR-584-5p-0.055}}$$

**Fig. 4:**
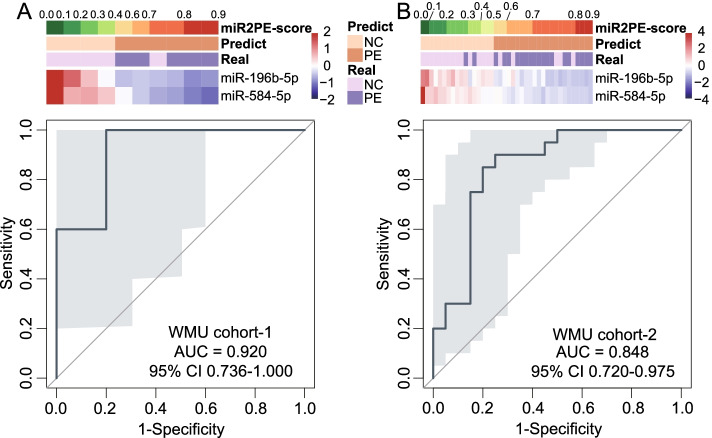
Establishment and validation of a miRNA signature for PE diagnosis (miR2PE-score). **A** Heatmap showing the expression pattern of the identified two miRNA biomarkers with the corresponding miR2PE-score, predict label and true label (above panel), and ROC for the performance of the miR2PE-score (below panel) in WMU cohort 1. **B** Heatmap showing the expression pattern of the identified two miRNAs with the corresponding miR2PE-score, predict label and true label (above panel), and ROC for the performance of the miR2PE-score (below panel) in WMU cohort 2. ROC, receiver operating characteristic curve; PE, preeclampsia

When miR2PE-score was applied to WMU cohort 1, the miR2PE-score and predicted labels of 5 PE placental samples and 5 normal placental samples were calculated and visualized in Fig. 4A. In WMU cohort 1, with the risk cutoff point of 0.5, five of five PE samples (100% sensitivity) and four of five normal samples (80.0% specificity) were correctly classified with an overall accuracy of 90.0% (nine of ten). The AUROC was 0.920 (95% confidence interval (CI) 0.736–1.000; Fig. 4A) and the area under the precision–recall curve (AUC-PR) was 0.919 (Additional file 1: Fig. S1A). To preliminarily verify cross-platform compatibility of miR2PE-score in PE diagnosis, the miR2PE-score was subsequently tested based on qRT-PCR data from WMU cohort 2. Figure 4B shows the expression levels of the two identified miRNAs, miR2PE-scores, and predicted labels and true labels of 20 PE and 20 control samples in WMU cohort 2. As expected, the miR2PE-score still works well in the qRT-PCR data and successfully identified 33 of 40 samples with an overall accuracy of 82.5%, an AUROC of 0.848 (95% CI 0.720–0.975; Fig. 4B), and PR-AUC of 0.815 (Additional file 1: Fig. S1B). These results initially confirmed that the miR2PE-score is reliable and compatible.

### Independent validation of the miR2PE-score in external multicenter cohorts

**Fig. 5:**
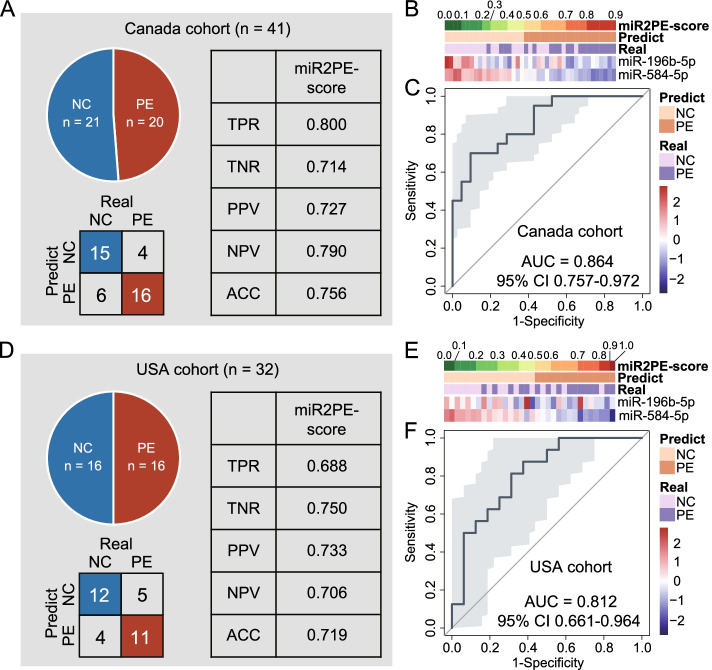
Independent validation of the miR2PE-score in external multicenter cohorts. A summary of the samples used to validate the performance of miR2PE-score to classify PE versus normal samples and the confusion matrix in the Canada cohort (**A**) and the USA cohort (**D**). TPR, true positive rate; TNR, true negative rate; PPV, positive predictive value; NPV, negative predictive value; ACC, overall accuracy. A heatmap of the expression pattern of identified two miRNA biomarkers with the corresponding miR2PE-score and predicted label and true label in the Canada cohort (**B**) and the USA cohort (**E**). The ROC for the performance of the miR2PE-score in the Canada cohort (**C**) and the USA cohort (**F**). ROC, receiver operating characteristic curve

To further evaluate the cross-platform compatibility and multinational robustness of the miR2PE-score, we tested the performance of the miR2PE-score in two external cohorts, including the Canada cohort with the miRNA sequencing platform and the USA cohort with the microarray platform. In the Canada cohort, with the same formula and the risk cutoff (0.5), 16 of 20 PE samples (80.0% sensitivity) and 15 of 21 normal samples (71.4% specificity) were correctly classified by the miR2PE-score with an overall accuracy of 75.6% (31 of 41; Fig. 5A). The two identified miRNA biomarkers, miR2PE-scores, and predicted labels and true labels of placental tissue samples in the Canada cohort are shown in Fig. 5B. As shown in Fig. 5C, the AUROC was 0.864 (95% CI 0.757–0.972; Fig. 5C) and the AUC-PR was 0.874 (Additional file 1: Fig. S2A). In the USA cohort, the miR2PE-score successfully identified 11 of 16 PE samples (68.8% sensitivity) and 12 of 16 normal samples (75.0% specificity) with an overall accuracy of 71.9% (23 of 32; Fig. 5D). The two identified miRNA biomarkers, miR2PE-scores, and predicted labels and true labels of placental tissue samples in the USA cohort are shown in Fig. 5E. Similarly, in the USA cohort, the miR2PE-score demonstrated an AUROC of 0.812 (95% CI 0.661–0.964; Fig. 5F) and an AUC-PR of 0.786 (Additional file 1: Fig. S2B). These results further confirmed that the predictive performance of miR2PE-score is reliable and universal across different technology platforms and countries.

### Prospective validation of miR2PE-score as a non-invasive biomarker in whole blood samples from two independent cohorts

**Fig. 6:**
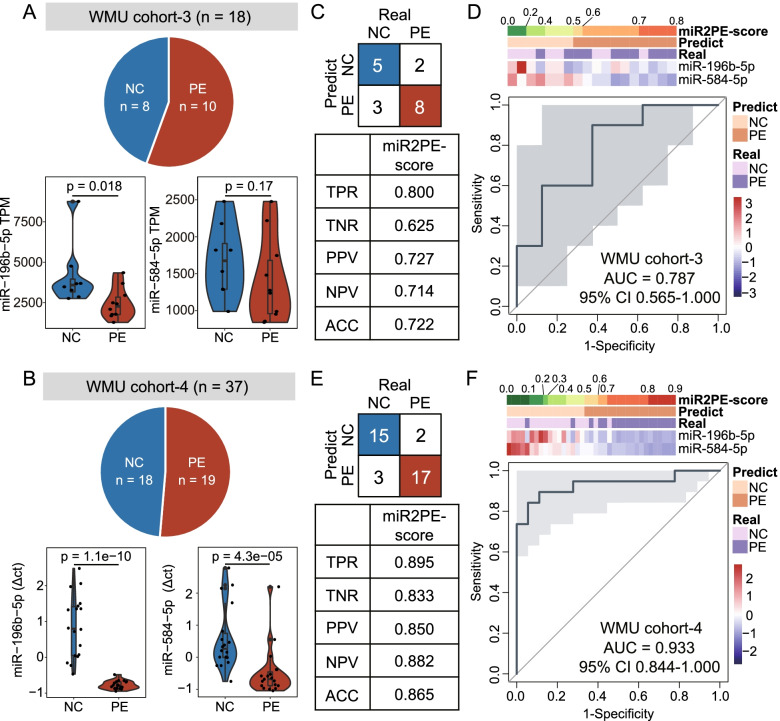
Performance evaluation of the miR2PE-score as a non-invasive biomarker in independent whole blood samples. A summary of the samples used to validate the performance of the miR2PE-score, and a boxplot showing expression levels of two miRNA biomarkers in WMU cohort 3 (**A**) and WMU cohort 4 (**B**). Horizontal lines: median values. The statistical analysis was performed using the Mann**–**Whitney *U* test. The confusion matrix shows the number of samples that are correctly identified in WMU cohort 3 (**C**) and WMU cohort 4 (**E**). Heatmap of the expression pattern of two miRNA biomarkers with the corresponding miR2PE-score and predict label and true label in WMU cohort 3 (**D**) and WMU cohort 4 (**F**) and the ROC for the performance of the miR2PE-score in WMU cohort 3 (**D**) and WMU cohort 4 (**F**). TPR, true positive rate; TNR, true negative rate; PPV, positive predictive value; NPV, negative predictive value; ACC, overall accuracy

To further examine whether the miR2PE-score can be detected in blood samples and used as a potential minimally invasive diagnostic biomarker, the expression levels of two identified miRNA biomarkers in peripheral blood leukocytes from WMU cohort 3 (including 10 PE and 8 normal samples) and WMU cohort 4 (including 19 PE and 18 normal samples) were analyzed with small RNA sequencing and qRT-PCR, respectively. As shown in Fig. 6A and B, two miRNA biomarkers (*miR-196b-5p* and *miR-584-5p*) revealed lower expression levels in peripheral blood leukocytes from PE patients than in healthy controls in both WMU cohort 3 and WMU cohort 4, which is consistent with observations from placental tissue-based validation. Using the miR2PE-score, 8 of 10 PE samples (80.0% sensitivity) and 5 of 8 normal samples (62.5% specificity) in WMU cohort 3 were correctly classified with an overall accuracy of 72.2% (13 of 18; Fig. 6C). The two identified miRNAs, miR2PE-scores, and predicted labels and true labels of placental tissue samples in WMU cohort 3 are shown in Fig. 6D. The AUROC was 0.787 (95% CI 0.565–1.000; Fig. 6D) and the AUC-PR was 0.823 (Additional file 1: Fig. S3A). In WMU cohort 4, 17 of 19 PE samples (89.5% sensitivity) and 15 of 18 normal samples (83.3% specificity) were correctly classified by the miR2PE-score with an overall accuracy of 86.5% (32 of 37; Fig. 6E). The two identified miRNAs, miR2PE-score, and predicted labels and true labels of placental tissue samples in WMU cohort 4 are shown in Fig. 6F. The AUROC was 0.933 (95% CI 0.844–1.000; Fig. 6F) and the AUC-PR was 0.953 (Additional file 1: Fig. S3B). Taken together, the above results suggest that miR2PE-score could serve as a minimally invasive diagnostic biomarker for the diagnosis of PE in the clinic.

### Performance comparison of the miR2PE-score with previously published miRNA biomarkers

**Fig. 7:**
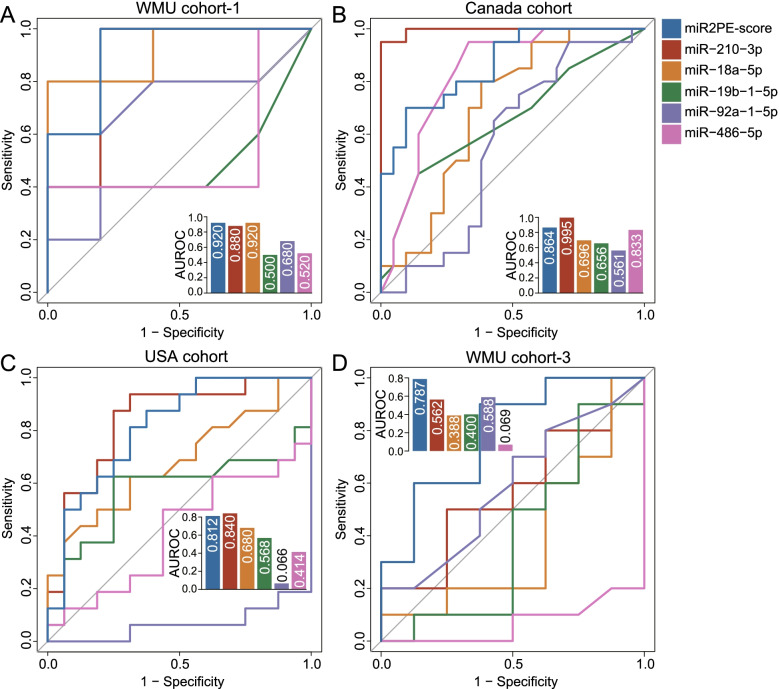
Performance comparison of the miR2PE-score with five recently published miRNA biomarkers to detect PE. ROCs are shown to compare performances of the miR2PE-score and five miRNAs in **A** WMU cohort 1, **B** the Canada cohort, **C** the USA cohort, and **D** WMU cohort 3

The performance of the miR2PE-score was further compared with five recently proposed PE miRNA biomarkers (*miR-210-3p*, *miR-19b-1-5p*, *miR-92a-1-5p*, *miR-486-5p*, and *miR-18a-5p*) in different cohorts. As shown in Fig. 7A–C, the miR2PE-score and *miR-210-3p* performed very well in all three placental tissue-based cohorts compared with the other four previously published miRNA biomarkers. However, the miR2PE-score achieved the best predictive performance in blood-based WMU cohort 3 compared with the other five previously published miRNA biomarkers (Fig. 7D). These results demonstrated that the miR2PE-score holds great potential to become a more effective and robust non-invasive biomarker for the early diagnosis of PE.

## Discussion

Preeclampsia is a pregnancy-specific disease with hypertension and proteinuria as the primary clinical manifestations and systemic arterial spasm as the main pathological features, affecting 2–4% of pregnancies [33, 34]; it poses a serious threat to the health of mothers and newborns. Prophylactic use of low-dose aspirin before the 16th gestational week appears to benefit pregnant women at high risk of preeclampsia from a perspective of preeclampsia prevention [35]. However, the clinical signs and symptoms of PE are non-specific and therefore pose a challenge to the early detection of women at the highest risk of developing PE. Although conventional diagnostic assessments for preeclampsia, such as blood pressure, proteinuria, lactate dehydrogenase, and other laboratory indicators, have been widely used, there is a lack of accurate testing criteria for preeclampsia. Their diagnostic performance may be affected by multiple clinical variables, such as race, body mass index, maternal comorbidities and/or obstetrical history, and unsatisfactory sensitivity and specificity [36, 37].

A growing body of evidence has showed that placental miRNAs play important functional roles in regulating placental pathophysiological processes and affecting adverse pregnancy outcomes [38–41]. The release of microRNAs from the placenta mainly occurs from the villous trophoblast cells, indicating that circulating microRNAs could serve as promising markers for monitoring trophoblast and placental function [42, 43]. Although abnormal expressions of circulating miRNAs have been found in pregnant women with preeclampsia, unfortunately, the clinical significance and predictive value of a few miRNAs have been systematically investigated and studied in PE diagnosis and outcomes [44–46]. Furthermore, a comparative study also indicated the unsatisfactory performance of potential miRNA markers previously reported in PE detection [47]. Therefore, the search for clinically viable non-invasive miRNA biomarkers for early detection of PE is still of utmost importance.

In the present study, we performed a high-throughput sequencing analysis for miRNAome and conducted a genome-wide screening for potential miRNA biomarkers. We identified five significantly differentially expressed miRNAs, and among which two miRNAs (*miR-196b-5p* and *miR-584-5p*) were finally selected by considering the association between the expression pattern and PE-associated clinical characteristics and qRT-PCR validation in tissues. From a functional viewpoint, the target genes of these two miRNA biomarkers have been shown to be involved in PE pathogenesis. Furthermore, a recent study by Mi et al. in PE models in vitro and in vivo revealed the pathological roles of the *BHLHE40*/*miR-196a-5p*/*SNX16* axis in PE [48]. The dysregulated expression of *miR-584* has also been observed in a previous placental microRNA expression study [49]. These existing pieces of knowledge further supported the critical roles of these two miRNA biomarkers identified in our study in PE pathogenesis, but their clinically applicable values in PE diagnosis remain questionable.

Therefore, we established a diagnostic signature (miR2PE-score) that comprised *miR-196b-5p* and *miR-584-5p* for the early detection of PE. To reduce the small sample bias of the maximum likelihood (ML) estimators of the logistic regression model, Firth’s bias-reduced logistic regression was introduced to fit the miR2PE-score. Firth’s bias-reduced logistic regression method is suitable for the problem of complete data separation caused by the extremely low number of cases. Furthermore, the miR2PE-score produced a scale of 0.0–1.0, which is beneficial to clinically risk probability assessment. Although the miR2PE-score was developed based on miRNA sequencing data, the performance of the miR2PE-score was also validated by qRT-PCR data, highlighting the clinical cost-effectiveness and cross-platform compatibility. It is important to note that the miR2PE-score was developed and validated primarily in our retrospective Chinese cohorts. Therefore, the miR2PE-score was further tested in external multicenter cohorts comprised of non-Asian races and also showed high diagnostic performance similar to that of our retrospective Chinese cohorts. These independent cohorts from different centers were not integrated to form a cohort. Therefore, we did not do batch correction and normalization. Each cohort is used individually to validate the expression patterns of these two miRNA biomarkers and further evaluate the performance and robustness of the miR2PE-score.

Considering potential clinical application of the miR2PE-score in true clinical settings, we expanded the evaluation of the miR2PE-score as a non-invasive liquid biopsy assay to prospectively collected plasma cohorts. Although expression levels of two miRNAs in the miR2PE-score were measured using different technical methods (miRNA sequencing and qRT-PCR), the miR2PE-score still demonstrated effectiveness in distinguishing PE from healthy subjects. Blood collection was performed before the cesarean leading to potential gestational age-matched in control subjects and PE patients, which may introduce variation in data, and the scoring may not be accurate in the clinic when evaluating patients at different gestational ages. Therefore, we also examine the diagnostic performance of the miR2PE-score after adjusting for gestational ages through logistic regression, which demonstrated that the miR2PE-score still is a significant predictive factor even after adjusting for gestational ages (Additional file 1: Fig. S4). Furthermore, we showed that the miR2PE-score performed significantly better than any previously published miRNA biomarkers in blood-based cohorts and achieved the best and most stable predictive performance compared to other parallel methods across different cohorts (Additional file 1: Fig. S5). However, a potential limitation of our study is that although we tested the miR2PE-score in cross-platform retrospective and prospective multicenter cohorts covering small cohort sizes, further validation should be warranted in population-based cohort studies.

## Conclusions

In conclusion, our study developed and validated a novel and robust blood-based miRNA signature for the early detection of PE in cross-platform retrospective and prospective multicenter cohorts. Investigations into non-invasive diagnostic performance in population-based prospective cohort studies warrant clinical transformation application in the future.

## Supplementary Information

**Additional file 1: Fig. S1.** Precision-Recall curve for the performance of the miR2PE-score in the WMU cohort-1 (A) and WMU cohort-2 (B). **Fig. S2.** Precision-Recall curve for the performance of the miR2PE-score in the Canada cohort (A) and USA cohort (B). **Fig. S3.** Precision-Recall curve for the performance of the miR2PE-score in the WMU cohort-3 (A) and WMU cohort-4 (B). **Fig. S4.** Forest plots of adjusted regression coefficients by gestational ages. **Fig. S5.** Performance comparison of the miR2PE-score with other parallel methods to detect PE.

## Data Availability

Raw small RNA-seq data generated during this study has been deposited in the Gene Expression Omnibus (GEO) database (https://www.ncbi.nlm.nih.gov/geo/, GSE206988) [50]. All public data are available from the GEO database (https://www.ncbi.nlm.nih.gov/geo/) under accession numbers GSE114349 [27] and GSE84260 [28].

## Abbreviations

AUROC: Area under the receiver operating characteristic curve
FDR: False discovery rate
GO: Gene Ontology
KEGG: Kyoto Encyclopedia of Genes and Genomes
NGS: Next-generation sequencing
PE: Preeclampsia
PlGF: Placental growth factor
qRT-PCR: Quantitative reverse transcription polymerase chain reaction
sEng: Soluble endoglin
sFlt-1: Fms-like tyrosine kinase-1
