# Supplementary material for: A blood-based miRNA signature for early non-invasive diagnosis of preeclampsia
Source: BMC Med. 2022 Sep 13;20:303. doi: 10.1186/s12916-022-02495-x (PMC9469544; doi:10.1186/s12916-022-02495-x)
Supplement: Supplementary file 1 — Additional file 1: Fig. S1. Precision-Recall curve for the performance of the miR2PE-score in the WMU cohort-1 (A) and WMU cohort-2 (B). Fig. S2. Precision-Recall curve for the performance of the miR2PE-score in the Canada cohort (A) and USA cohort (B). Fig. S3. Precision-Recall curve for the performance of the miR2PE-score in the WMU cohort-3 (A) and WMU cohort-4 (B). Fig. S4. Forest plots of adjusted regression coefficients by gestational ages. Fig. S5. Performance comparison of the miR2PE-score with other parallel methods to detect PE. [file 12916_2022_2495_MOESM1_ESM.pdf]

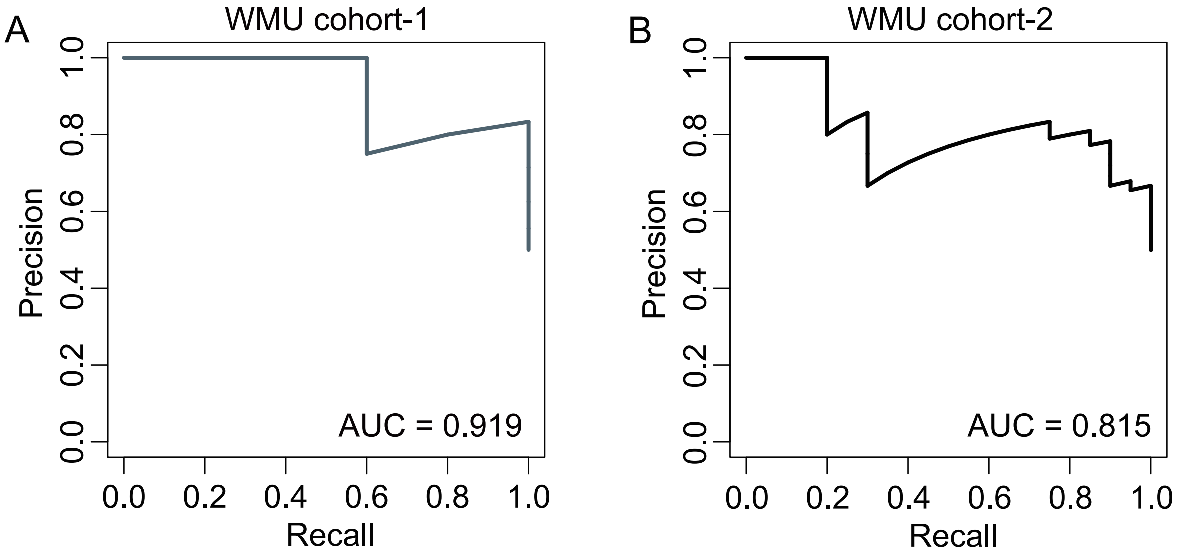

Fig.S1. Precision-Recall curve for the performance of the miR2PE-score in the WMU cohort-1 (A) and WMU cohort-2 (B).

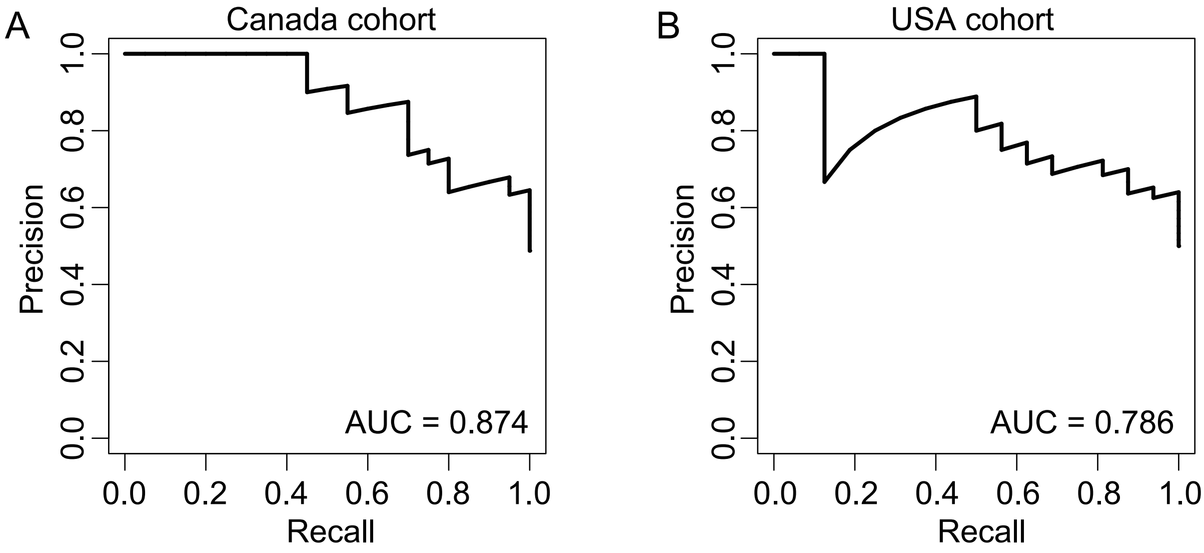

Fig.S2. Precision-Recall curve for the performance of the miR2PE-score in the Canada cohort (A) and USA cohort (B).

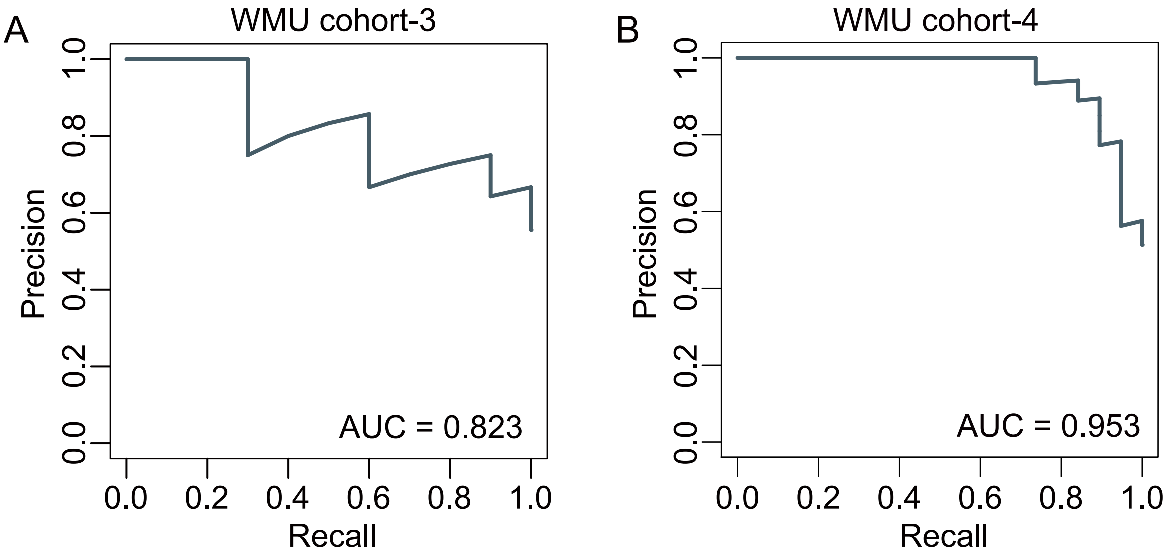

Fig.S3. Precision-Recall curve for the performance of the miR2PE-score in the WMU cohort-3 (A) and WMU cohort-4 (B).

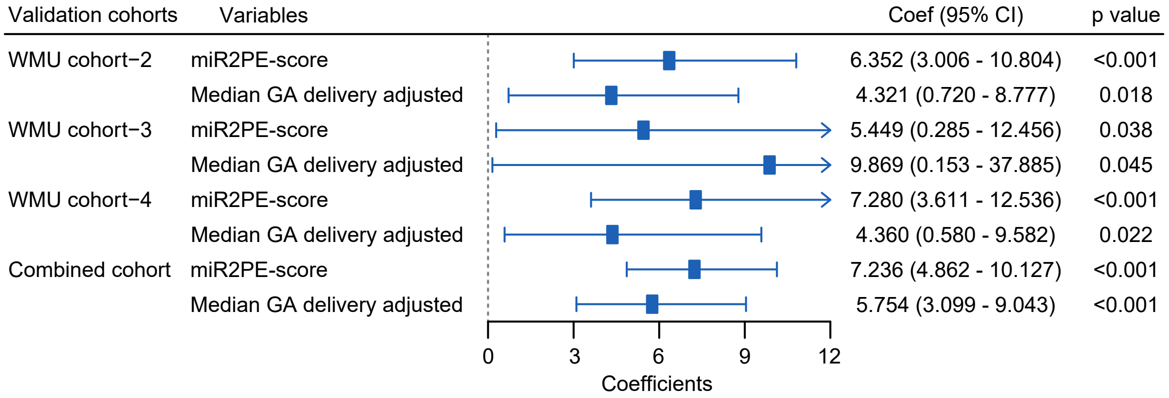

Fig.S4. Forest plots of adjusted regression coefficients by gestational ages.

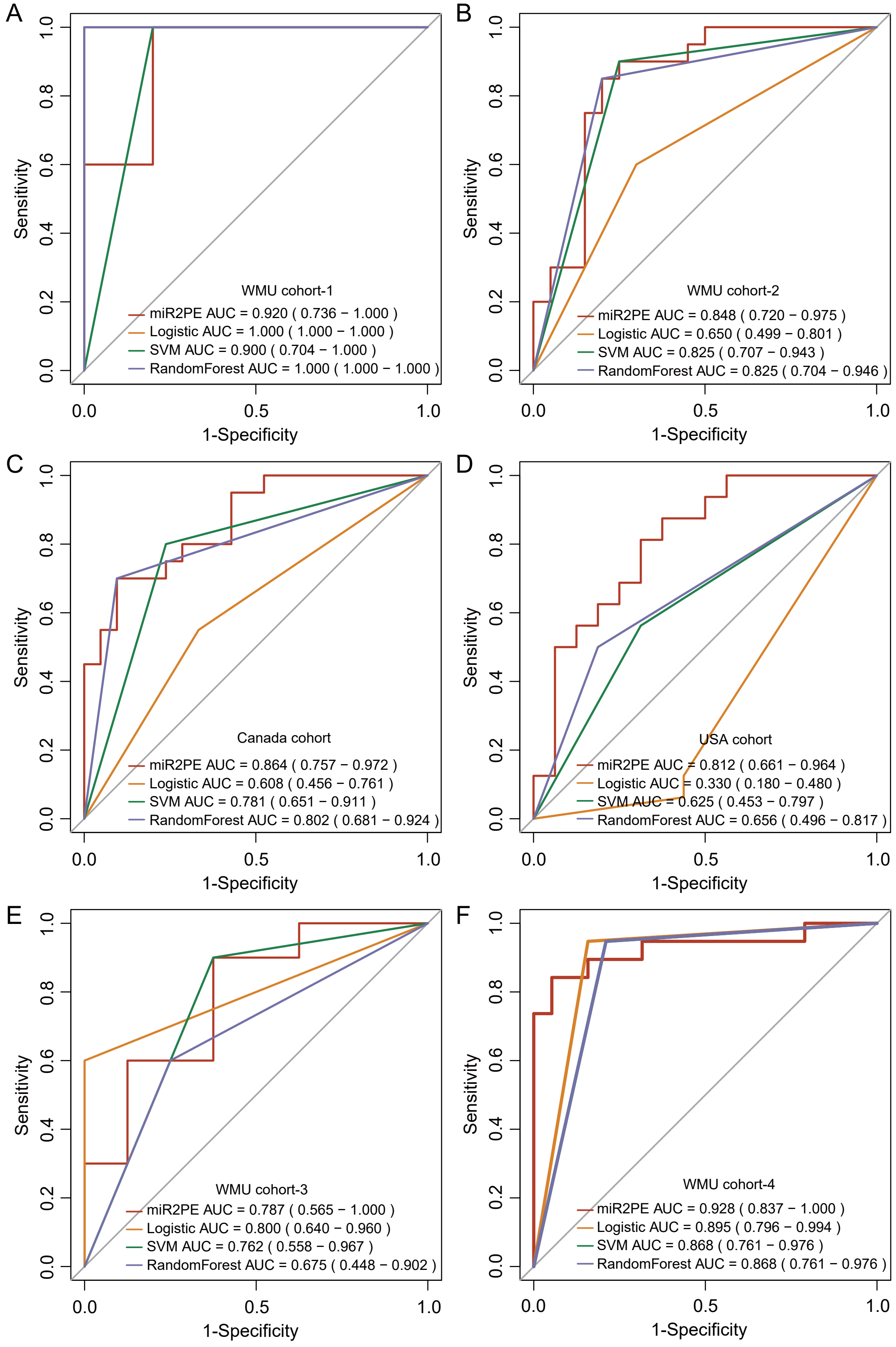

Fig.S5. Performance comparison of the miR2PE-score with other parallel methods to detect PE.
